# Progress in the study of autophagy-related proteins affecting resistance to chemotherapeutic drugs in leukemia

**DOI:** 10.3389/fcell.2024.1394140

**Published:** 2024-06-03

**Authors:** Meng Li, Jing Li, Shiming Zhang, Linghan Zhou, Yuanyuan Zhu, Shen Li, Qiong Li, Junjie Wang, Ruipeng Song

**Affiliations:** ^1^ Nursing Department, The Third People’s Hospital of Henan Province, Zhengzhou, China; ^2^ Department of Pathophysiology, Sepsis Translational Medicine Key Laboratory of Hunan Province, Xiangya School of Medicine, Central South University, Changsha, Hunan, China; ^3^ Clinical College, Xiamen Medical University, Xiamen, Fujian, China; ^4^ Rehabilitation Department, Henan Institute of Massage, Luoyang, Henan, China; ^5^ Nursing Department, Xinxiang Medical University, Xinxiang, China; ^6^ Plastic Surgery, The Third People’s Hospital of Henan Province, Zhengzhou, China; ^7^ Endocrinology Department, The Third People’s Hospital of Henan Province, Zhengzhou, China

**Keywords:** autophagy-associated proteins, leukaemia, drug tolerance, chemotherapeutic drugs, review

## Abstract

Leukemia is a life-threatening malignant tumor of the hematopoietic system. Currently, the main treatment modalities are chemotherapy and hematopoietic stem cell transplantation. However, increased drug resistance due to decreased sensitivity of leukemia cells to chemotherapeutic drugs presents a major challenge in current treatments. Autophagy-associated proteins involved in autophagy initiation have now been shown to be involved in the development of various types of leukemia cells and are associated with drug resistance. Therefore, this review will explore the roles of autophagy-related proteins involved in four key autophagic processes: induction of autophagy and phagophore formation, phagophore extension, and autophagosome formation, on the development of various types of leukemias as well as drug resistance. Autophagy may become a promising therapeutic target for treating leukemia.

## 1 Introduction

Leukemia is a disease in which the normal physiological activity of the bone marrow is impeded by the overproduction of immature white blood cells in the bone marrow and blood tissues, resulting in abnormal hematopoietic function and destruction of organs. It is a life-threatening malignant tumor of the hematopoietic system. Clinically, there are four main types of leukemia, namely, acute myeloid leukemia (AML), acute lymphoblastic leukemia (ALL), chronic lymphocytic leukemia (CLL), and chronic myelogenous leukemia (CML). Currently, the more common treatments for leukemia are chemotherapy and hematopoietic stem cell transplantation. Recently, the advent of targeted drugs has mitigated several leukemias to some extent. For example, 80%–90% of patients with acute promyelocytic leukemia (APL) are cured following the use of trans-retinoic acid and arsenic trioxide (Lo-Coco et al., 2013), and ponatinib, a third-generation drug of tyrosine kinase inhibitor (TKI) therapy, offers a treatment option for CML patients with the Bcr-Abl T315I mutation (Breccia et al., 2018). Some subtypes of leukemia remain poorly treated, especially AML. AML is primarily characterized by complex and dynamic genomic instability, AML patients under 60 years of age have a better prognosis and cure rates approaching 35%–40%, only 5%–15% of patients over 60 years of age are in remission (Döhner et al., 2015). AML is prone to relapse and drug resistance, which may be due to mutations in genes associated with epigenetic modifications (TET2, IDH1 and IDH2, DNMT3A, ASXL1, WT1, EZH2), genes associated with dysregulation of DNA repair (TP53, NPM1), and genes associated with defects in cell cycle inhibition and differentiation (NPM1, CEBPA, TP53, and GATA2).

Cellular autophagy is a process by which cells encapsulate parts of their cytoplasm and organelles in a double membrane structure and fuse them with lysosomes for degradation and recycling. The purpose of cellular autophagy is to eliminate excess or defective cellular components, renew cellular structures, provide energy and raw materials, and protect cell and tissue function. Cellular autophagy is a conserved cellular process that is associated with cell and tissue regeneration, aging, and diseaseTranslated with www.DeepL.com/Translator (free version). Which is categorized into three main types: macroautophagy, microautophagy, and chaperone-mediated autophagy. All three of these different forms of autophagy ultimately deliver the phagocytosed material to the lysosome for degradation and recycling (Yang and Klionsky, 2010). The process of autophagy is broadly divided into the following four stages: induction of autophagy, assembly and formation of autophagosomes, fusion of autophagosomes with lysosomes, and degradation and recirculation of autophagosome contents (Li X. et al., 2020). The discovery of autophagy-related proteins has led to a better understanding of the molecular mechanisms of autophagy regulation. For example, the ULK1 complex is involved in the formation of phagocytic vesicles and controls the extension of phagocytic vesicles and the formation of autophagosomes (Mizushima, 2010), and Beclin1, a core subunit in the PI3KC3 complex, interacts with another core subunit, VPS34, to activate VPS34 kinase activity to regulate the size and number of autophagosomes (Backer, 2008).

**FIGURE 1:**
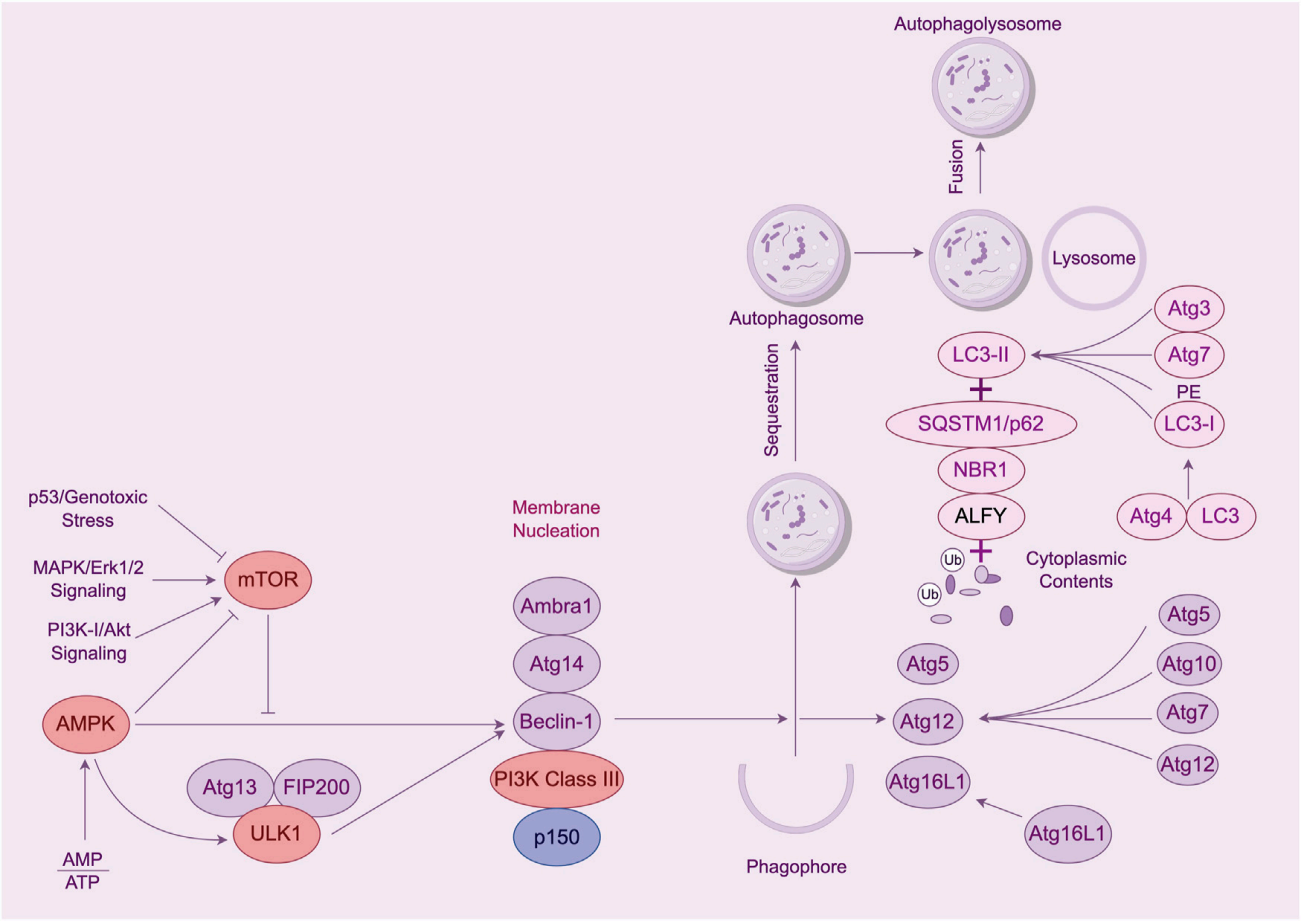
The process of cellular autophagy and its key proteins. Note: The mTOR kinase is a key molecule in autophagosome induction. mTOR activation pathways such as Akt and MAPK signaling pathways inhibit autophagy, and pathways that negatively regulate mTOR, such as AMPK and p53 signaling pathways, promote autophagy. mTOR kinases are the only core proteins of the autophagosome signaling pathway that have a serine/threonine kinase activity. Prior to autophagy lysosome assembly autophagy signaling is mediated through the activation of the ULK complex composed of ULK1, FIP200, and Atg13. The ULK1 complex *in vivo* serves as a bridge connecting the upstream nutrient or energy-receptor mTOR and AMPK to the formation of downstream autophagosomes. AMPK activates the phosphorylation of ULK1 thereby facilitating the assembly of the ULK1 complex to initiate autophagy. Class Lll PI3K complex includes Beclin-1, Atg14, p150, and Ambra1, all of which are required for the induction of autophagy. The Atg genes control autophagosome formation through the Atg12-Atg5 and LC3-ll complexes. Atg12 binds to Atg5 in a ubiquitin-like reaction that requires Atg7 and Atg10, which are E1-and E2-like enzymes, respectively. coupling. The Atg12-Atg5 linker then reacts noncovalently with Atg16 to form a larger complex. The C-terminal cup of LC3/Atg8 is proteolytically cleaved by the Atg4 protease to generate the cytoplasmic LC3-l.LC3-l is also coupled to phosphatidylethanolamine (PE) in a ubiquitin-like reaction, a reaction that requires both Atg7 and Atg3. A lipid form of LC3, known as LC3-ll, adsorbs to the autophagosome membrane. This links LC3 to autophagic vesicles. The presence of LC3 in autophagosomes and its conversion to the low-migratory form, LC3-ll, is used as an indicator of the onset of autophagy.

Autophagy is a process by which cells degrade themselves. See Figure 1 and Table 1. It is important for balancing sources of energy and coping with nutritional stress during critical periods of development. Also autophagy and autophagy-related proteins are involved in the development of leukemia (Piya et al., 2016; Hu et al., 2018; Pei et al., 2018). This article focuses on a review of the genes and proteins involved in the autophagy process in association with leukemia.

**TABLE 1:** The process of autophagy and its involved proteins.

Stage of autophagy	Key players/Proteins	Function of each protein	References
Autophagy Initiation	ULK1 complex (ULK1, ATG13, FIP200, ATG101), mTORC1	ULK1: Initiates autophagy by phosphorylation of the autophagy machinery; phosphorylated by AMPK or mTORC1 ATG13:Mediates the junction of the interaction between ULK1 and FIP200; enhances ULK1 kinase activity, phosphorylated by mTORC1 FIP200: Component of the ULK1 complex; scaffolding role (ULK1/2 and ATG13)	Mizushima (2010), Jung et al. (2009), Hara et al. (2008), Ganley et al. (2009), Lee et al. (2020)
Phagophore Formation	Beclin-1, VPS34, ATG14, VPS15 (PI3K complex)	Beclin-1: Promotes PI3K1-C1 complex assembly; regulates VSP34; ULK1/AMPK phosphorylation sites; promotes autophagy VPS34: Promotes PI3KC3-C1 complex formation, a phosphorylation site for ULK1, and stabilizes the ULK1 complex ATG14: ULK1 phosphorylation site; targets PI3K3-C1 to autophagosome formation sites; contributes to phagosome expansion VPS15: Serine/threonine kinase; VPS34 regulatory protein	Backer (2008), Hu et al. (2021), Peng et al. (2013), Can et al. (2011)
Phagophore Extension	ATG12–ATG5-ATG16L1 complex, LC3/ATG8 (and PE conjugation)	ATG12–ATG5-ATG16L1 complex: ATG5 directly binds membranes, and this membrane binding is negatively regulated by ATG12 but activated by ATG16; membrane binding of the ATG12-ATG5-ATG16L1 complex is required to efficiently promote ATG8 esterification (conversion of LC3-l to LC3-ll) LC3/ATG8: Exists in two forms, LC3-1 and LC3-II; involved in the formation of autophagosome membranes, binds to PE on the surface of autophagosome membranes, and can be used as a labeling molecule for autophagosomes	Dooley et al. (2014), Tanida et al. (2004), Piya et al. (2016)
Autophagosome Formation	ATG2, WIPI1-4, ATG9	ATG2:ATG2 is part of the ATG9/ATG12-WIPI complex, which is essential for ATG9 recruitment to expand extended autophagosomes WIPI1-4:WIPI 1-4 is part of the ATG2-WIPI complex, which is important for ATG9 recruitment to autophagosomes, binds to PI3P, which is required for retrograde transport of ATG9, and to ATG2 ATG9: Transmembrane protein; interacts with ATG2-WlPl complex; shuttles between PAS and peripheral organelles to deliver lipids/factors during phagophore expansion, and self-interaction	Jang et al. (2017b), Dooley et al. (2014), Li et al. (2021)

## 2 Autophagy initiation and phagophore formation

Autophagy can be induced by a variety of intra- and extracellular factors. For example, mild uncoupling of oxidative phosphorylation can be influenced by mitochondria-targeted cations thereby inducing autophagy (Lyamzaev et al., 2018), and autophagy can be induced when there are fewer mTOR kinases localized to the lysosome and the activity of mTORC1 is reduced (Hertel et al., 2022). Stimulated by these intra- and extracellular factors, the pre-autophagic structure (PAS), as a structure that can recruit autophagy-associated proteins (Atg), recruits almost all autophagy-associated proteins. Among them, ULK1 complex and PI3K complex target PAS in a hierarchical manner and participate in the formation and assembly of autophagosomes (Mizushima et al., 2011). The ULK1 complex is mainly composed of ATG13, FIP200, and ATG101. The complex further binds to itself to generate the PAS scaffold complex. Subsequently, the PIK3 complex coalesces onto the PAS, binds to the ATG13 interaction of the PAS via ATG14L, and participates in the formation of phagolysosomes. The ULK1 protein and Beclin-1 protein play a key role in the autophagy process of leukemia.

### 2.1 ULK1 protein

ULK1 is a serine/threonine protein kinase that plays a crucial role in the initiation of autophagy. In most cells, the absence of ULK1 disrupts autophagy. In one study, downregulation of ULK1 expression led to the inhibition of autophagy (Chan et al., 2007). During the onset of autophagy, ULK1 binds to three proteins, ATG13, FIP200 and ATG101, to form a complex with each other (Hosokawa et al., 2009; Jung et al., 2009). This complex has a role in activating autophagy (Hara et al., 2008), where ATG13 or FIP200 increases the activity and stability of ULK1 (Ganley et al., 2009). The formation of a complex between ATG13 and FIP200 provides structural support for ULK1 and helps to maintain the stability of the complex to prevent its degradation. At the same time, ATG13 and FIP200 contribute to the subcellular localization of ULK1 and can directly regulate the activity of ULK1. ULK1 has been shown to be involved in the generation of its autophagy in many diseases, for example, in pancreatic cancer, NEDD4L can interact with ULK1 to reduce ULK1 expression to inhibit autophagy and mitochondrial metabolism, which in turn inhibits the survival of pancreatic cancer cells (Lee et al., 2020). Another study demonstrated that upregulation of ULK1 in Jurkat cells and CD4^+^ T cells after being infected by HIV induced autophagy for defense against HIV invasion (Wang et al., 2012). A growing number of studies have found that ULK1 can influence leukemia development by regulating autophagy in various types of leukemia cells. See Table 2.

**TABLE 2:** The role of proteins in autophagy in leukemia.

Protein	Mechanism of action in leukemia	Signaling pathways and related proteins	Specific types of leukemia	References
ULK1	Initiates autophagy, aiding leukemia cell survival in nutrient-poor conditions. Linked to chemoresistance and poor prognosis	AMPK activates ULK1 under stress; mTORC1 inhibits it under nutrient-rich conditions. Involved in the AMPK/mTOR signaling pathway	Acute Myeloid Leukemia (AML), Chronic Lymphocytic Leukemia (CLL)	Mizushima (2010), Jung et al. (2009), Jang et al. (2017a), Ianniciello et al. (2021), Ianniciello and Helgason (2022)
mTORC1	Promotes cell growth and proliferation by inhibiting autophagy. Upregulated activity is linked to therapy resistance	Regulates ULK1 through phosphorylation. Part of the PI3K/AKT/mTOR pathway	Acute Lymphoblastic Leukemia (ALL), AML	Ganley et al. (2009), He et al. (2016), Yu et al. (2020), Bosnjak et al. (2014))
TIGAR	Reduces ROS, shifts metabolism, indirectly modulating autophagy and contributing to resistance	Operates downstream of p53, affecting glycolysis and the pentose phosphate pathway	AML, particularly in relation to metabolic reprogramming	Hu et al. (2021), Li et al. (2021)
p62/SQSTM1	Links autophagy to the ubiquitin-proteasome system; its accumulation activates survival pathways, impacting proliferation and survival	Interacts with LC3 and ubiquitinated substrates; involved in NRF2 signaling pathway activation	AML, ALL, CLL, especially where autophagy is impaired	Hu et al. (2018), Yuan et al. (2015), Wang et al. (2023)

#### 2.1.1 Role of ULK1 protein on AML autophagy

Patients with AML are highly susceptible to developing resistance to chemotherapy drugs. In recent years, studies have found that ULK1 can induce autophagy production in AML to increase patient sensitivity to chemotherapeutic agents, thereby reducing drug resistance. FLT3 inhibitors can be used to target FLT3-ITD + AML, but acquired resistance occurs rapidly in most patients (Tarver et al., 2020). FLT3 inhibitors can overcome AML resistance to FLT3 inhibitors by inducing autophagy production through the AKT-mTORC1-ULK1 axis with the help of ATG3 (Koschade et al., 2022). Combination chemotherapy with cytarabine/anthracycline can lead to complete remission in some patients, but relapse associated with drug resistance remains a common cause of treatment failure. Anthracycline-based Zoerythromycin (DNR) can induce autophagy production through the AMPK-ULK1 signaling pathway, which inhibits DNR resistance thereby increasing DNR drug sensitivity in AML (Qiu et al., 2020). The AMPK-ULK1 signaling pathway can induce autophagy to increase the sensitivity of leukemia stem cells (LSC) to BET inhibitors in AML (Jang et al., 2017a; Jang et al., 2017b). Also, ULK1 can interact with proteins or genes to activate autophagy in AML. NPM1 mutations are the most common genetic alteration in AML. The most common type NPM1 mutation are type-A (NPM1-mA), which counts for70%-80% cases. NPM1-mA can neutralize ULK1 in AML. It also positively regulates ULK1 expression and maintains ULK1 stability. It was noted that NPM1-mA enhanced TRAF6-dependent ubiquitination and further maintained ULK1 stability through miR-146a, which effectively activated autophagy to promote AML cell survival (Tang et al., 2021). In another study, knockdown of ULK1 downregulated the MCL1 gene; damaging leukemia cells by impairing mitochondrial function and downregulating CD44-xCT, resulting in ROS mitigation of DNA damage and promotion of apoptosis (Bhattacharya et al., 2023). Caspase-3 is an important regulator of AML autophagy, and it can promote autophagy in AML cells by interacting with ULK1 (Man et al., 2017).

#### 2.1.2 Role of ULK1 protein on CML autophagy

Tyrosine kinase inhibitors (TKIs) are the mainstay of treatment for chronic myelogenous leukemia (CML) today. However, leukemia stem cells (LSC) that maintain tiny residual disease (MRD) foci will rely on basic metabolic processes to resist drug treatment (Ianniciello et al., 2021). Recent studies have found that inhibition of ULK1 expression in LSC can stress-induce LSC differentiation, causing it to be sensitive to TKI treatment (Ianniciello and Helgason, 2022). The increase in LSC sensitivity is driven by the inhibition of autophagy, increased mitochondrial respiration, and loss of quiescence caused by ULK1 deletion (Ianniciello et al., 2021). Imatinib, as the first targeted drug capable of inhibiting BCR-ABL kinase activity for the treatment of CML, is still resistant to imatinib in some CML patients (Druker et al., 2006). Resistance due to BCR-ABL point mutations is a major barrier to TKI treatment of CML. It has been demonstrated that BIIB021 can promote apoptosis in imatinib-resistant CML cells by inducing autophagy through the Akt-mTOR-ULK1 pathway (He et al., 2016). GCA was identified as a key factor regulating resistance to imatinib in CML. GCA promoted TRAF6 ubiquitination ligase allowing ubiquitination of ULK1 lys63, and the result of this ubiquitination activated autophagy in CML cells, which modulated CML resistance to imatinib (Han et al., 2019). Meanwhile, ULK1 can also affect CML resistance to imatinib by inducing autophagy through the ceRNA pathway. Circ-0009910 can regulate ULK1-induced autophagy via sponge miR-34a-5p thereby promoting CML resistance to imatinib (Cao et al., 2020).

#### 2.1.3 Role of ULK1 protein on other diseases autophagy

The advent of targeted therapies has led to a fundamental change in the treatment of chronic lymphocytic leukemia. MRT68921 has potent cytotoxicity against CLL cells as a ULK1 inhibitor that disrupts autophagy and causes cell cycle G2 blockade in CLL cells. Also, in combination with venetoclax, it enhanced cysteine enzyme-dependent cytotoxicity (Avsec et al., 2021). This suggests that autophagy inhibitors have some potential for the treatment of CLL. Similarly, enhanced autophagy helps leukemia. Myelodysplastic syndromes (MDS) have a very high risk of transformation into AML (Corey et al., 2007), and increased expression of sperm-associated antigen 6 (SPAG6) has been detected in patients with AML transformed by MDS and in patients with new-onset AML (Steinbach et al., 2006). Upon knockdown of SPAG6, the AMPK/mTOR/ULK1 signaling pathway in SKM-1 cells was activated thereby inducing autophagy, which ultimately led to increased apoptosis in SKM-1 cells (Zhang M. et al., 2020). This shows the potential of activating autophagy to treat leukemia. ULK1 can also interact with plant extracts to play a role in acute leukemia. Pomegranate, the main phenolic compound in pomegranate peel, andrographis paniculata can induce autophagy production in acute leukemia by up-regulating ULK1 expression (Subkorn et al., 2021). In another study, sesquiterpenes likewise upregulated ULK1 expression open to activate cellular autophagy (Deesrisak et al., 2021). In both studies, activation of autophagy improved the effectiveness of treating leukemia.

### 2.2 Beclin-1 protein

Beclin one is a novel Bcl-2-homology (BH)-3 structural domain protein, one of the first autophagy effectors identified (Liang et al., 1999). Beclin-1 functions as a metamorphic regulator of the PI3KC3 complex. In the initiation of cellular autophagy, Beclin-1 often forms a complex with PI3KC3, the second important autophagy signaling complex that continues to induce the onset of autophagy after the role of the ULK1 complex (Funderburk et al., 2010). In cancer autophagy, the interaction of Beclin-1 with JAK2 is triggered by IL-6, which allows JAK2 to phosphorylate Beclin-1 at the Y333 site. This process promotes the formation of the PI3KC3 complex thereby activating autophagy in colon cancer (CRC) cells (Hu et al., 2021). Beclin-1 can be activated by the PI3K/Atk signaling pathway and plays a role in inducing autophagy in hepatocellular carcinoma cells by being regulated by BCL2L10 (He et al., 2019). In myocardial ischemia-reperfusion injury, ischemia preconditioned (IPC)-treated rat cells showed suppression of Beclin-1-dependent excessive autophagy, which reduced myocardial ischemia-in-perfusion injury-induced cell death (Peng et al., 2013). From the above studies, it can be found that Beclin-1 is involved in the development of autophagy in different diseases, and there is no exception in leukemia. More and more studies have shown that Beclin-1-induced autophagy plays a role in leukemia.

#### 2.2.1 Role of Beclin-1 protein on ALL autophagy

The development of drug resistance remains a major challenge in the treatment of acute lymphoblastic leukemia (ALL). How to improve the sensitivity of ALL patients to drugs is the key to treating ALL. Glucocorticoids are widely cited for the treatment of ALL, but unintermittent use can lead to the development of resistance. It has been reported that roughly 20% of children with ALL are resistant to glucocorticoids, and even up to 70% of children with recurrent ALL are resistant to glucocorticoids (Inaba and Pui, 2010). miRNAs have a wide range of roles in leukemia, among which, miR-145 enhances the sensitivity of ALL cell lines to glucocorticoids, which is achieved by promoting the expression of Beclin-1 and Bax genes and inhibiting the expression of Bcl-2 genes to induce autophagy and apoptosis production (Long et al., 2020). The first-generation tyrosine kinase inhibitor (TKI) imatinib (IM) can be used not only for the treatment of CML, but has also been widely used in patients with Ph(+) ALL. Imatinib resistance in Ph(+) ALL cells is mediated by the hnRNPK/Beclin-1 signaling pathway. hnRNPK can bind to Beclin-1 in Ph(+) ALL, and upregulation of hnRNPK promotes the generation of autophagic vesicles in Ph(+) ALL cells, which enhances the resistance of Ph(+) ALL cells to imatinib (Zhang J. et al., 2022). Bortezomib is a proteasome inhibitor that promotes its therapeutic effects when combined with autophagy inhibitors. This is due to the fact that bortezomib promotes the formation of Beclin-1/PI3KC3 complex and activates autophagy in ALL cells, which ultimately leads to a decrease in the toxic effect of bortezomib on ALL cells (Wang et al., 2015).

Bafilomycin A1, known for its specific inhibition of the V-ATPase, plays a critical role in autophagy by preventing the acidification of various organelles, including lysosomes. This inhibition disrupts the fusion between autophagosomes and lysosomes, a key step in the degradation of autophagic cargo, making it a valuable tool for studying autophagic flux. At high doses, it is commonly used to block this fusion or inhibit lysosomal activity crucial for late-stage autophagy (Lee et al., 2020).

Recent research has demonstrated the dual role of bafilomycin A1 in targeting both autophagy and apoptosis pathways. In pediatric B-cell acute lymphoblastic leukemia (B-ALL), low concentrations of bafilomycin A1 were shown to effectively induce apoptosis in primary cells from patients, highlighting its potential as an anticancer agent. Moreover, toxicity evaluation in mice indicated that doses up to 10 mg/kg were well tolerated, with higher doses showing signs of liver toxicity (Lee et al., 2020).

This specificity in inhibiting V-ATPase and its consequential blockade of autophagosome-lysosome fusion, coupled with its ability to activate apoptosis, underscores the therapeutic potential of bafilomycin A1 in cancer treatment. By manipulating autophagy pharmacologically, bafilomycin A1, along with other autophagy inhibitors, could improve clinical outcomes in leukemia and other cancers by enhancing the activity of anticancer agents (Lee et al., 2020). Beclin-1 can also bind to Bcl-2, which is induced by bafilomycin A1 (Bafilomycin A1), further inhibiting autophagy and promoting apoptosis in ALL cells (Yuan et al., 2015).

The evidence highlights a critical linkage between Beclin-1-mediated autophagy and the efficacy of therapeutic agents in acute lymphoblastic leukemia (ALL), particularly influencing drug tolerance. This connection underscores the necessity for nuanced treatment strategies that consider autophagy’s dual role in enhancing drug sensitivity and resistance, pointing toward the potential of autophagy modulation as a complementary approach in ALL therapy.

#### 2.2.2 Role of Beclin-1 protein on other diseases autophagy

Beclin-1’s influence extends beyond autophagy regulation, impacting therapeutic outcomes and drug resistance mechanisms in acute promyelocytic leukemia (APL) and chronic myeloid leukemia (CML). In APL, the autophagy pathway activated by Beclin-1 has shown an inhibitory effect on the therapeutic efficacy of bortezomib, a proteasome inhibitor. Specifically, Beclin-1 knockdown in APL cells led to reduced autophagy, enhancing bortezomib’s apoptotic effect on NB4 cell lines (Jiang et al., 2021). This suggests that autophagy modulation might enhance the sensitivity of APL cells to bortezomib, providing a strategic approach to overcome drug resistance.

The interaction between Beclin-1 and the BCR-ABL oncogene in CML unveils another layer of complexity in autophagy’s role in leukemia. BCR-ABL, known for its constitutive tyrosine kinase activity, promotes leukemogenesis and drug resistance. Beclin-1’s engagement with BCR-ABL not only triggers autophagy but also targets BCR-ABL for degradation via autophagic mechanisms, facilitated by the co-localization with p62/SQSTM1 in autolysosomes. This process potentially diminishes the oncogenic influence of BCR-ABL and enhances the efficacy of tyrosine kinase inhibitors (TKIs) (Huang et al., 2019; Yu et al., 2020).

The strategic degradation of BCR-ABL through Beclin-1 mediated autophagy suggests a novel therapeutic pathway to mitigate TKI resistance, a prevalent challenge in CML treatment. Enhancing autophagy or specifically augmenting the Beclin-1 and BCR-ABL interaction could serve as a therapeutic strategy to decrease BCR-ABL levels, thus improving TKI treatment outcomes.

This intricate relationship between Beclin-1 and oncogenic proteins in leukemia underlines the critical role of autophagy in cancer biology, offering insights into novel therapeutic targets. Further investigation into Beclin-1’s specific mechanisms of action and its interactions with oncogenes like BCR-ABL could unlock new avenues for treatment strategies aimed at leveraging autophagy modulation to combat drug resistance in leukemia.

For a deeper understanding, the following references provide comprehensive insights.

- The inhibitory effect of Beclin-1 on bortezomib in APL cells suggests a nuanced approach to autophagy modulation could improve therapeutic outcomes.
- The interaction between Beclin-1 and BCR-ABL in CML highlights the potential of targeting autophagy pathways to enhance TKI efficacy and overcome drug resistance (Huang et al., 2019; Yu et al., 2020).

Exploring these pathways offers a promising direction for enhancing leukemia treatment efficacy and addressing the challenge of drug resistance through the modulation of autophagy.

## 3 Phagophore extension and autophagosome formation

The ATG-related protein family plays a major role in the extension of phagolysosomes as well as the formation of autophagosomes. This process is mainly mediated by the ATG12-ATG5 coupling system and the ATG8-LC3 coupling system. ATG12-ATG5 will form an oversized complex with ATG16 (Dooley et al., 2014), and this complex will eventually bind to treated LC3, lipidating LC3 (Tanida et al., 2004), allowing phagophore extension and closure. Eventually, in the presence of the two coupled systems, a closed bilayer membrane structure is formed. The mature autophagosome thus arises (Suzuki et al., 2013).

### 3.1 ATG-related proteins

Autophagy-related (ATG) proteins are central to the autophagy process, a critical cellular mechanism for degrading and recycling cytoplasmic components to maintain cellular health and respond to stress. Among the array of ATG proteins, ATG5, ATG7, and ATG10 play pivotal roles in the conjugation processes essential for the formation and maturation of autophagosomes. ATG5 is part of a conjugate with ATG12, facilitated by ATG7 (acting as an E1-like enzyme) and ATG10 (an E2-like enzyme), crucial for the expansion of the autophagosome membrane. The ATG8-LC3 system, another ubiquitin-like conjugation mechanism, further assists in the autophagosome’s expansion and cargo recruitment, with LC3 being a well-recognized marker for autophagy.

Leukemia, particularly acute myeloid leukemia (AML), demonstrates the complexity of autophagy’s role in cancer. In AML, the autophagic process, mediated by ATG proteins, may offer a double-edged sword—promoting cell survival in some contexts while enabling therapeutic targeting in others. The dysregulation of autophagy, either through enhanced or diminished activity of ATG proteins, can influence leukemia cell fate, affecting responses to chemotherapy and targeted therapies. This highlights the potential of targeting autophagy pathways as a therapeutic strategy in leukemia, underscoring the need for further research to understand the nuanced roles of ATG proteins in mediating autophagy within this specific disease context.

This background underscores the significance of ATG proteins not only in the fundamental process of autophagy but also in the broader implications for disease progression and treatment strategies in leukemia.

The formation process of mature autophagosomes is mainly associated with two coupling systems, ATG12-ATG5 and ATG8-LC3. Meanwhile this process is involved by various ATG proteins, for example, ATG7 and ATG10 act as E1-and E2-like enzymes, respectively, mediating the coupling process of ATG12 and AGT5 (Nakatogawa, 2013). In studies of solid tumors, ATG5 is involved in drug resistance of gastric cancer cells by regulating autophagy (Ge et al., 2014); in glioblastoma (GBM) ATG5 can be mediated by PAK1 to produce phosphorylation to promote autophagosome production to achieve hypoxia-induced autophagy (Feng et al., 2021); ATG7 can be modulated by Celastroal, an active substance extracted from Ranunculus ternatus in rectal cancer, which is achieved by the inhibition of Nur77 expression by Celastroal, and the simultaneous elevation of ATG7 expression promoted autophagy in rectal cancer cells (Zhang W. et al., 2022). Drug resistance in gastrointestinal mesenchymal stromal tumor (GIST) is associated with the activation of autophagy. It was noted that circ-CCS could downregulate ATG10 by targeting miR-197-3P, and the autophagy-promoting effect of circ-CCS on mesenchymal tumor cells was reversed after knockdown of miR-197-3p (Sui et al., 2022). The studies listed above illustrate that ATG-related proteins play a role in solid cancers in relation to autophagy. In leukemia, especially acute myeloid leukemia, ATG-related proteins play a role by mediating autophagy.

#### 3.1.1 Role of ATG-related protein on AML autophagy

ATG-related proteins are widely used in drugs for the treatment of AML. Decitabine (DAC) not only has the effect of inhibiting the methylation of DNA, but also promotes the formation of autophagosomes in AML cells by down-regulating the expression of TIGAR, which leads to the upregulation of ATG3, ATG5, LC3, and Beclin-1 proteins and the downregulation of p62 (Li et al., 2021). ATG7 can be regulated by EVI1 to induce autophagy in myeloid leukemia. This modulation protects myeloid leukemia cells and reduces the efficacy of drugs (Niu et al., 2020). Cytarabine is susceptible to resistance in the treatment of AML (Dombret and Gardin, 2016). However, inhibition of cellular autophagy *in vitro* can increase the sensitivity of AML cells to cytarabine (Bosnjak et al., 2014). It has been suggested that miR-143 can enhance cytotoxicity induced by cytarabine by targeting ATG7 and ATG2B-dependent autophagy (Zhang H. et al., 2020). Treatment with cytarabine activates leukemia initiating cell (LIC) activity. When ATG7 defects can promote elevated mitochondrial activity, reactive oxygen species production, and apoptosis, this enhances the therapeutic effect of cytarabine (Sumitomo et al., 2016). Knockdown of ATG7 promotes autophagy in AML cells and inhibits autophagy and chemoresistance, which contributes to an increase in overall survival of AML patients (Piya et al., 2016). ATG5-dependent autophagy promotes the development of AML, while knockdown of ATG5 improves AML sensitivity to chemotherapeutic agents (Liu et al., 2016; Wang et al., 2023), ATG5 also mediates the potential differentiation capacity of AML MSCs and the cell cycle distribution, which leads to autophagy and improves AML chemosensitivity (Li Y. et al., 2020).

#### 3.1.2 Role of ATG-related protein on other diseases autophagy

The role of ATG-associated protein-mediated autophagy in CML can be manifested in imatinib resistance. lncRNA OIP5-ASI can promote CML autophagy-associated imatinib resistance through the miR-30e-5p/ATG12 axis (Dai et al., 2021). Imatinib can also promote Beclin-1 and ATG5 expression to induce CML autophagy (Can et al., 2011). Currently, in studies on myelodysplastic syndromes, ATG3-mediated autophagy was found to have an inhibitory effect on the survival of MDS cells, and upregulation of ATG3 expression in MDS cells promoted Akt-mTOR-dependent autophagy, which inhibited the proliferation as well as promoted apoptosis of MDS cells (Wang et al., 2014; Zhuang et al., 2016).

## 4 Conclusion

Autophagy as an evolutionarily conserved catabolic process in cells. Various autophagy-related proteins involved in autophagy play key roles in the development of various types of leukemias. The proteins mainly play a role in the two processes of autophagy initiation and mature autophagosome formation in leukemia, mainly ULK1 complex, Beclin-1 protein, and ATG-related proteins. Autophagy mediated by these proteins plays a role in the treatment of different types of leukemia as well as drug resistance.

Today, the availability of several drugs and new treatments has prolonged or saved the lives of many leukemia patients. However, the resistance of leukemia patients to chemotherapeutic drugs has become the main reason for the refractory treatment and relapse of leukemia. It is certain that more and more studies have confirmed that autophagy mediated by autophagy-associated proteins can reduce the resistance of leukemia cells to certain chemotherapeutic drugs in order to increase the sensitivity of the cells to the drugs. This suggests that autophagy can promote the treatment of leukemia. Therefore, in the near future, preventive promotion of autophagy inducers or inhibitors in combination with modulation of autophagy activity, based on leukemogenesis and different phenotypes, could serve as a potential anti-leukemia therapy.

In summary, autophagy mediated by autophagy-associated proteins has different degrees of effects on the treatment of various leukemias, mainly in the fight against drug resistance. However, the mechanism of resistance to many therapeutic drugs and autophagy is still unclear, and further studies are needed to understand how autophagy contributes to the development and treatment of leukemia, and to provide more evidence on how autophagy mediated by autophagy-associated proteins can improve the sensitivity of various types of leukemias to therapeutic drugs.
